# Supplementary material for: Minimum Infective Dose of a Lumpy Skin Disease Virus Field Strain from North Macedonia
Source: Viruses. 2020 Jul 16;12(7):768. doi: 10.3390/v12070768 (PMC7411612; doi:10.3390/v12070768)
Supplement: Supplementary file 1 [file viruses-12-00768-s001.pdf]

viruses

MDPI

Supplementary Materials: Minimum infective dose of a Lumpy skin disease virus field strain from North Macedonia

Janika Wolff, Kiril Krstevski, Martin Beer and Bernd Hoffmann

Table S1. Pan Capripox real-time qPCR results of different sample materials after inoculation with different infective doses of LSDV-strain "Macedonia2016".

	Genome Copies/µL Template at Different Days Post Inoculation										
**Group A 3.8 × 10^2 $CCID_{50}$/animal**	−1/3	5	7	10	11	12	13	14	17	21	28
**R/279_EDTA blood**	no Cq	no Cq	no Cq	no Cq	-	no Cq	-	no Cq	no Cq	no Cq	no Cq
**R/279_serum**	no Cq	no Cq	no Cq	no Cq	-	0.15	-	no Cq	no Cq	no Cq	no Cq
**R/279_nasal swab**	no Cq	no Cq	no Cq	no Cq	-	no Cq	-	0.27	0.14	no Cq	no Cq
**R/278_EDTA blood**	no Cq	no Cq	no Cq	0.44	-	no Cq	-	111.00	no Cq	0.26	no Cq
**R/278_serum**	no Cq	no Cq	no Cq	no Cq	-	no Cq	-	7.01	no Cq	no Cq	no Cq
**R/278_nasal swab**	no Cq	no Cq	no Cq	no Cq	-	no Cq	-	no Cq	0.14	no Cq	no Cq
**R/280_EDTA blood**	no Cq	no Cq	no Cq	1.09	-	43.60	1.38	†	†	†	†
**R/280_serum**	no Cq	no Cq	0.92	0.33	-	3.05	0.97	†	†	†	†
**R/280_nasal swab**	no Cq	no Cq	no Cq	no Cq	-	3.24	158.00	†	†	†	†
**R/824_EDTA blood**	no Cq	no Cq	no Cq	no Cq	-	no Cq	-	no Cq	no Cq	67.20	†
**R/824_serum**	no Cq	no Cq	no Cq	no Cq	-	no Cq	-	no Cq	no Cq	1.44	†
**R/824_nasal swab**	no Cq	no Cq	no Cq	no Cq	-	0.55	-	no Cq	0.54	3.73	†
**R/915_EDTA blood**	no Cq	no Cq	no Cq	no Cq	-	no Cq	-	no Cq	0.70	no Cq	no Cq
**R/915_serum**	no Cq	no Cq	no Cq	0.78	-	no Cq	-	0.40	no Cq	no Cq	no Cq
**R/915_nasal swab**	no Cq	no Cq	no Cq	no Cq	-	no Cq	-	no Cq	no Cq	no Cq	no Cq
**R/203_EDTA blood**	no Cq	no Cq	no Cq	no Cq	-	no Cq	-	no Cq	no Cq	no Cq	no Cq
**R/203_serum**	no Cq	no Cq	no Cq	no Cq	-	no Cq	-	no Cq	no Cq	no Cq	no Cq
**R/203_nasal swab**	no Cq	no Cq	no Cq	no Cq	-	no Cq	-	no Cq	no Cq	no Cq	no Cq
**Group B 2.4 × 10^4 $CCID_{50}$/animal**											
**R/888_EDTA blood**	no Cq	no Cq	no Cq	15.40	0.36	†	†	†	†	†	†
**R/888_serum**	no Cq	no Cq	no Cq	2.45	2.00	†	†	†	†	†	†

**R/888_nasal swab**	no Cq	-	no Cq	no Cq	no Cq	†	†	†	†	†	†
**R/857_EDTA blood**	no Cq	no Cq	no Cq	no Cq	-	no Cq	-	no Cq	no Cq	no Cq	no Cq
**R/857_serum**	no Cq	no Cq	no Cq	no Cq	-	no Cq	-	no Cq	no Cq	no Cq	no Cq
**R/857_nasal swab**	no Cq	no Cq	no Cq	no Cq	-	0.20	-	no Cq	0.49	no Cq	no Cq
**R/891_EDTA blood**	no Cq	no Cq	no Cq	258.00	-	0.20	-	0.17	no Cq	2.71	no Cq
**R/891_serum**	no Cq	no Cq	no Cq	no Cq	-	no Cq	-	no Cq	no Cq	2.30	no Cq
**R/891_nasal swab**	no Cq	no Cq	no Cq	no Cq	-	0.03	-	no Cq	no Cq	no Cq	no Cq
**R/829_EDTA blood**	no Cq	no Cq	no Cq	19.10	-	0.25	0.17	†	†	†	†
**R/829_serum**	no Cq	no Cq	no Cq	0.82	-	0.36	no Cq	†	†	†	†
**R/829_nasal swab**	no Cq	no Cq	no Cq	no Cq	-	no Cq	no Cq	†	†	†	†
**R/984_EDTA blood**	no Cq	no Cq	0.48	3.72	-	154.00	41.10	†	†	†	†
**R/984_serum**	no Cq	no Cq	no Cq	1.10	-	1.43	5.31	†	†	†	†
**R/984_nasal swab**	no Cq	no Cq	no Cq	no Cq	-	36.10	4.34	†	†	†	†
**R/990_EDTA blood**	no Cq	no Cq	no Cq	0.48	-	0.01	-	0.10	no Cq	no Cq	no Cq
**R/990_serum**	no Cq	no Cq	no Cq	no Cq	-	no Cq	-	no Cq	no Cq	no Cq	no Cq
**R/990_nasal swab**	no Cq	no Cq	no Cq	no Cq	-	no Cq	-	0.17	0.36	no Cq	no Cq
**Group C 3.3 × 10^6 $CCID_{50}$/animal**											
**R/277_EDTA blood**	no Cq	no Cq	no Cq	3.21	-	1.01	1.27	†	†	†	†
**R/277_serum**	no Cq	no Cq	no Cq	0.49	-	0.32	0.37	†	†	†	†
**R/277_nasal swab**	no Cq	no Cq	no Cq	no Cq	-	0.17	0.15	†	†	†	†
**R/283_EDTA blood**	no Cq	no Cq	0.87	265.00	136.00	†	†	†	†	†	†
**R/283_serum**	no Cq	no Cq	no Cq	1.64	1.70	†	†	†	†	†	†
**R/283_nasal swab**	no Cq	no Cq	no Cq	1.37	4.02	†	†	†	†	†	†
**R/284_EDTA blood**	no Cq	no Cq	51.40	8.96	-	10.20	-	0.92	no Cq	no Cq	no Cq
**R/284_serum**	no Cq	no Cq	0.38	2.04	-	2.10	-	0.17	no Cq	0.34	no Cq
**R/284_nasal swab**	no Cq	no Cq	no Cq	no Cq	-	no Cq	-	no Cq	no Cq	no Cq	no Cq
**R/921_EDTA blood**	no Cq	no Cq	51.40	8.96	-	10.20	-	0.92	no Cq	no Cq	no Cq
**R/921_serum**	no Cq	no Cq	0.38	2.04	-	2.10	-	0.17	no Cq	0.34	no Cq
**R/921_nasal swab**	no Cq	no Cq	no Cq	no Cq	-	no Cq	-	no Cq	no Cq	no Cq	no Cq

**R/841_EDTA blood**	no Cq	1.53	7.05	1.85	10.10	†	†	†	†	†	†
**R/841_serum**	no Cq	no Cq	no Cq	1.45	2.14	†	†	†	†	†	†
**R/841_nasal swab**	no Cq	no Cq	no Cq	no Cq	no Cq	†	†	†	†	†	†
**R/989_EDTA blood**	no Cq	no Cq	8.34	41.20	3.25	†	†	†	†	†	†
**R/989_serum**	no Cq	0.91	no Cq	9.18	1.95	†	†	†	†	†	†
**R/989_nasal swab**	no Cq	no Cq	no Cq	4.49	257.00	†	†	†	†	†	†
**Group D 3.8 × 10^7 $CCID_{50}$/animal**											
**R/276_EDTA blood**	no Cq	no Cq	no Cq	5320.00	-	0.01	-	no Cq	37.70	no Cq	62.80
**R/276_serum**	no Cq	no Cq	no Cq	10.90	-	0.77	-	no Cq	0.37	no Cq	no Cq
**R/276_nasal swab**	no Cq	no Cq	no Cq	0.26	-	no Cq	-	no Cq	no Cq	no Cq	no Cq
**R/282_EDTA blood**	no Cq	1.68	no Cq	0.10	-	no Cq	-	no Cq	no Cq	no Cq	no Cq
**R/282_serum**	no Cq	no Cq	0.33	no Cq	-	no Cq	-	no Cq	no Cq	no Cq	no Cq
**R/282_nasal swab**	no Cq	no Cq	no Cq	1.62	-	no Cq	-	2.70	0.27	no Cq	no Cq
**R/981_EDTA blood**	no Cq	41.60	9.75	13.50	62.30	†	†	†	†	†	†
**R/981_serum**	no Cq	no Cq	1.08	2.96	4.59	†	†	†	†	†	†
**R/981_nasal swab**	no Cq	no Cq	no Cq	883.00	5880.0	†	†	†	†	†	†
**R/860_EDTA blood**	no Cq	41.60	9.75	13.50	62.30	†	†	†	†	†	†
**R/860_serum**	no Cq	no Cq	1.08	2.96	4.59	†	†	†	†	†	†
**R/860_nasal swab**	no Cq	no Cq	no Cq	883.00	5880.0	†	†	†	†	†	†
**R/893_EDTA blood**	no Cq	no Cq	no Cq	5.74	-	0.97	-	0.53	no Cq	no Cq	no Cq
**R/893_serum**	no Cq	no Cq	no Cq	0.69	-	no Cq	-	0.21	no Cq	no Cq	no Cq
**R/893_nasal swab**	no Cq	no Cq	no Cq	no Cq	-	3.28	-	0.18	0.07	no Cq	no Cq
**R/988_EDTA blood**	no Cq	16.10	173.00	841.00	382.00	†	†	†	†	†	†
**R/988_serum**	no Cq	1.35	14.60	87.30	327.00	†	†	†	†	†	†
**R/988_nasal swab**	no Cq	no Cq	1.10	438.00	11700.00	†	†	†	†	†	†

The genome copy numbers per μL template are presented. For calculation of the genome copy number per ml sample material, the listed genome copies must be multiplied by a factor of 400. † displays animal was euthanized before sampling day, - means sample not taken.

Table S2. Pan Capripox real-time qPCR results of different tissue samples of cattle after inoculation with different infective doses of LSDV-strain "Macedonia2016".

Cattle		Cervical Lymph Node	Medestinal Lymph Node	Mesenterial Lymph Node	Spleen	Liver	Lung	Neck (Skin Nodules)	Udder Mirror (Skin Nodules)	Pox-Like Lesions Lung
**Group A—3.8 × 10^2 $CCID_{50}$/animal**	**R/279**	no Cq	no Cq	no Cq	no Cq	no Cq	no Cq	no Cq	n.t.	n.t.
	**R/278**	no Cq	no Cq	no Cq	no Cq	no Cq	no Cq	n.t.	n.t.	n.t.
	**R/280**	96.45	no Cq	no Cq	no Cq	0.25	0.42	251000.0	5445.0	n.t.
	**R/824**	136.50	no Cq	no Cq	1.98	0.56	75.40	199500.0	714500.0	1665000.0
	**R/915**	0.78	no Cq	no Cq	no Cq	no Cq	no Cq	n.t.	n.t.	n.t.
	**R/203**	no Cq	no Cq	no Cq	no Cq	no Cq	no Cq	n.t.	n.t.	n.t.
**Group B—2.4 × 10^4 $CCID_{50}$/animal**	**R/888**	15.50	no Cq	no Cq	no Cq	no Cq	2.12	144500.0	38350.0	n.t.
	**R/857**	no Cq	no Cq	no Cq	no Cq	no Cq	no Cq	n.t.	n.t.	n.t.
	**R/891**	no Cq	no Cq	no Cq	no Cq	no Cq	no Cq	n.t.	n.t.	n.t.
	**R/829**	1.00	no Cq	no Cq	no Cq	no Cq	no Cq	n.t.	n.t.	n.t.
	**R/984**	8.20	no Cq	no Cq	no Cq	0.43	5.56	25400.0	53700.0	n.t.
	**R/990**	no Cq	no Cq	no Cq	no Cq	no Cq	no Cq	n.t.	n.t.	n.t.
**Group C—3.3 × 10^6 $CCID_{50}$/animal**	**R/277**	no Cq	no Cq	no Cq	no Cq	no Cq	4.82	1255000.0	3750000.0	36800.0
	**R/283**	41.55	0.59	0.66	no Cq	no Cq	8.22	113500.0	580000.0	n.t.
	**R/284**	no Cq	no Cq	no Cq	no Cq	no Cq	no Cq	no Cq	n.t.	10105.0
	**R/921**	no Cq	no Cq	no Cq	no Cq	no Cq	no Cq	n.t.	n.t.	n.t.
	**R/841**	35.75	4.02	no Cq	no Cq	no Cq	8.18	91850.0	114050.0	n.t.
	**R/989**	24.45	35.90	no Cq	no Cq	no Cq	1.61	84150.0	n.t.	6320.0
**Group D—3.8 × 10^7 $CCID_{50}$/animal**	**R/276**	no Cq	no Cq	no Cq	no Cq	no Cq	no Cq	n.t.	n.t.	5940.00
	**R/282**	no Cq	no Cq	no Cq	no Cq	no Cq	no Cq	n.t.	n.t.	n.t.
	**R/981**	24.40	0.09	no Cq	no Cq	no Cq	42.70	56900.0	400000.0	n.t.
	**R/860**	no Cq	no Cq	no Cq	no Cq	no Cq	33.50	n.t.	n.t.	n.t.
	**R/893**	no Cq	no Cq	no Cq	no Cq	no Cq	no Cq	no Cq	no Cq	1590.00
	**R/988**	2555.00	68700.0	2.77	1400.00	105.90	148000.0	144000.0	577000.0	1940000.0

The genome copy numbers per μL template are presented. For calculation of the genome copy number per g tissue material, the listed genome copies must be multiplied by a factor of 4000. n.t. displays sample not taken.
